# Effects of different dietary energy levels on growth performance, meat quality and nutritional composition, rumen fermentation parameters, and rumen microbiota of fattening Angus steers

**DOI:** 10.3389/fmicb.2024.1378073

**Published:** 2024-05-06

**Authors:** Kaihao Chen, Yanghaoer Shui, Ming Deng, Yongqing Guo, Baoli Sun, Guangbin Liu, Dewu Liu, Yaokun Li

**Affiliations:** ^1^Herbivore Laboratory, College of Animal Science, South China Agricultural University, Guangzhou, China; ^2^National Joint Engineering Research Center, South China Agricultural University, Guangzhou, China; ^3^Guangdong Key Laboratory of Agricultural Animal Genomics and Molecular Breeding, South China Agricultural University, Guangzhou, China

**Keywords:** Angus, integrated net energy, rumen fermentation parameters, unsaturated fatty acids, rumen microbiota

## Abstract

This study investigates the effects of varying energy levels in diets on Black Angus steers, focusing on growth performance, muscle composition, rumen microbial community, and their interrelationships. Twenty-seven Black Angus steers, aged approximately 22 months and weighing 520 ± 40 kilograms, were randomly divided into three groups: low-energy (LE), medium-energy (ME), and high-energy (HE). Each group consisted of nine individuals. The steers were fed diets with energy levels of 6.657 MJ/kg (LE), 7.323 MJ/kg (ME), and 7.990 MJ/kg (HE) following a 14-day pre-feeding period, with a subsequent 90-day main experimental phase. After the 90-day feeding period, both the HE and ME groups exhibited significantly higher average daily weight gain (ADG) compared to the LE group (*p* < 0.05). The feed-to-weight ratios were lower in the HE and ME groups compared to the LE group (*p* < 0.05). The HE group showed significantly higher crude fat content in the longissimus dorsi muscle compared to the LE group (*p* < 0.05), with total fatty acid content in the muscle surpassing that in the ME and LE groups (*p* < 0.05). As dietary energy levels increased, the diversity of the rumen microbial community decreased (*p* < 0.05), and significant differences in bacterial community structure were observed between the LE and HE groups (*p* < 0.05). The results suggest that higher dietary energy levels enhance growth performance and alter muscle composition in Black Angus steers, while also influencing the rumen microbial community. This study contributes to understanding optimal dietary strategies for finishing Angus cattle to improve beef quality, economic returns, and the development of standardized production procedures.

## 1 Introduction

Angus cattle have been extensively distributed across various countries around the globe. This breed is renowned for its exceptional muscularity, superior average daily gain, and elevated levels of beef fat content (Albertí et al., 2008). Angus cattle are characterized by their early maturity, exceptional growth rate, exceptional eye muscle, and outstanding yield traits (Chambaz et al., 2003). Typically, Angus cattle undergo prolonged fattening, and upon reaching 18 months of age, they are transitioned to a high-energy diet. This strategy is often aimed at the premium consumer market, characterized by increased fattening costs, an extended cycle, and a high input–output ratio (Heffernan et al., 2021). Extensive research has demonstrated that the nutritional composition of feed significantly influences the deposition of fat within the muscle tissue. Fat deposition requires a substantial energy intake; when the energy needs for maintaining beef cattle are fulfilled, excess energy is then directed toward the development of muscle, bones, and fat accumulation. Administering high-energy diets enhances the net energy supply, boosting the intake of volatile fatty acids and glucose, which, in turn, promotes fat deposition in the muscle tissue (Wittek et al., 2018). The loin backfat content of Angus beef cattle is approximately 10%, reflecting a strong capacity for fat deposition in this breed (Greenwood et al., 2015).

During different growth and development stages, optimal levels of protein and energy in the diet can augment nutrient utilization efficiency and enhance production outcomes. The study by Wang et al. (2020) revealed significant increases in the daily weight gain and dry matter intake in fattened Hu lamb fed diets with elevated energy levels. Liu et al. (2023) found that, under conditions of high-concentrated feed, cattle exhibit a higher average daily weight gain. Yang et al. (2020) found that, during the fattening process of yaks, a high-energy diet led to improved feed efficiency and greater daily weight gain compared to a low-energy diet. A study by Wang et al. (2019) also demonstrated increased dry matter intake (DMI) and average daily gain (ADG) in the later stages of fattening with higher dietary energy levels. Enhancing feed intake while concurrently reducing feed costs can improve the economic efficiency of beef cattle production. A multitude of studies have indicated that, in ruminants, satiety is primarily determined by gastrointestinal fill and regulated by physical signals (Fernández-López et al., 2002; Palesty and Dudrick, 2003; Aiello et al., 2017; de Godoy, 2018; dela Peña et al., 2022). When not restricted by dry matter intake (DMI), cattle typically consume feed with a consistent energy level. A decrease in dietary energy levels typically leads to an increase in DMI (Krehbiel et al., 2006). In prepubertal heifers, gastrointestinal capacity is the primary factor influencing intake with low-energy diets (Davis Rincker et al., 2008).

Maintaining a stable rumen environment is critical for the productive efficiency of beef cattle. Rumen fatty acids contribute to 60–70% of the energy requirements of ruminant animals (Zhang et al., 2021). Changes in the nutritional composition of the diet can significantly affect the rumen environment, leading to alterations in the composition of rumen microorganisms and subsequently impacting microbial fermentation products. The study by Ge demonstrated that, in fattening sheep, the butyric acid level in the rumen fluid rises with increased daily dietary energy levels. Additionally, high-energy diets have been observed to reduce the richness of the microbial community (Ge et al., 2023). The study by Ahmad has shown that increased dietary energy levels significantly lower the pH value of rumen fluid in yaks. Simultaneously, significant increases in the concentrations of acetic acid, propionic acid, butyric acid, valeric acid, and total volatile fatty acids (VFAs) have been noted. While no significant differences were noted in rumen bacterial communities with increased dietary energy levels, the microbial diversity index was marginally higher in the low-energy group compared to the moderate-energy and high-energy groups (Ahmad et al., 2020). Liu et al. (2022) discovered that as the Metabolizable Energy to Nitrogen (ME:N) ratio decreases, the total concentration of rumen volatile fatty acids and the molar proportion of propionate increase, while the molar proportion of acetate and the acetate to propionate ratio decreases. Furthermore, variations in the ME:N ratio result in the changes in the composition of the rumen bacterial community.

This study, through feeding cattle diets with varying energy levels, aimed to investigate the effects of high-energy diets on Angus steers, focusing on aspects such as rumen fermentation, gastrointestinal microbiota, and meat quality. The comprehensive approach adopted is intended to identify the optimal dietary energy level, with the ultimate goal of enhancing the economic efficiency of Angus steer rearing.

## 2 Materials and methods

All experimental procedures and sample collection methods complied with the Regulation on the Administration of Laboratory Animals (CLI.2.293192, 2017 Revision, State Council, China) and were performed in accordance with the Institutional Animal Care and Use Committee of South China Agricultural University (approval no. 2018-P002).

### 2.1 Animal management, diets, and sampling

The study was conducted at the Angus beef cattle ecological farm of Meizhou Guangshunhai Food Co., Ltd. Twenty-seven Black Angus steers, aged approximately 22 months (±1) and with an average body weight of 520 ± 40 kg, were selected for the experiment. These Angus steers were randomly allocated into three treatment groups, each consisting of nine steers, in accordance with a single-factor completely randomized design. A pre-feeding period of 14 days was followed by a 90-day experimental phase. Throughout the experimental phase, each group received a total mixed ration (TMR) tailored to the specified dietary net energy for maintenance (Ne_mf_): 6.657 MJ/kg for the LE group, 7.323 MJ/kg for the ME group, and 7.990 MJ/kg for the HE group. TMR diets were formulated according to the Chinese Feed Composition and Nutritional Value Tables (31st edition) (Xiong et al., 2020). Feeding was conducted twice daily at 9:00 a.m. and 4:00 p.m., and water was available *ad libitum*. Uniform management was applied to all experimental cattle. Details regarding the composition and the nutritional content of the dietary ingredients are shown in Table 1.

**Table 1:** Composition and nutritional profile of the experimental diets (% dry matter).

Items	LE	ME	HE
Ingredients			
Corn	46.82	49.25	53
Soybean meal	8.99	9.08	8.99
Wheat bran	5.81	5.71	5.52
Sodium bicarbonate	0.66	0.66	0.66
Salt	0.66	0.66	0.66
^1^Premix	2.62	2.63	2.62
Rumen passing fat acid	0	2.53	4.68
Corn straw silage	34.46	29.49	23.88
Total	100	100	100
Nutrition level			
^2^NE_mf_ MJ/kg	6.657	7.323	7.99
CP	11.5	11.51	11.5
EE	2.87	5.4	7.61
Starch	36.24	38.02	40.74
NDF	33.64	30.41	26.81
ADF	18.61	16.51	14.14
Ca	0.48	0.47	0.46
P	0.44	0.43	0.42

^1^Beef cattle premix, per kilogram (based on dry matter): Copper 191 mg, Iron 1,200 mg, Manganese 1,393 mg, Selenium 9 mg, Vitamin A 250 KIU, Vitamin E 1500 IU, Vitamin B1 699 mg, Niacin 1,500 mg. ^2^The total net energy is a calculated value; all others are measured values.

Throughout the main trial, fresh samples of the TMR mixed diets were collected on days 1, 20, 40, 60, 80, and 90. Leftover feed was collected the following morning before feeding and was then dried in a 65°C oven to constant weight. Subsequently, the dried feed samples were ground, sieved through a 40-mesh sieve, and stored in polyethylene bags. Stomach fluid samples were collected from the cattle on days 1, 45, and 90 prior to the morning feeding. During each sampling, approximately 150 mL of stomach fluid was collected. After collection, stomach fluid pH was immediately measured using a pH meter (FE28-Standard, METTLER-TOLEDO Corporation), and samples were then transferred to 2 mL cryotubes for storage in liquid nitrogen. The remaining stomach fluid was filtered through four layers of sterile gauze, aliquoted into 20 mL centrifuge tubes, and centrifuged at 5,000 rpm for 15 min. Subsequently, the supernatant was stored at −20°C for further analysis of stomach fluid fermentation parameters and 16S DNA sequencing. On day 91 of the trial, three cattle from each of the LE, ME, and HE groups were randomly selected for slaughter. Before slaughter, the cattle underwent a 16-h fasting period and a 3-h water restriction period. After slaughter, samples were collected from the longissimus muscle between the left 12th and 13th rib intercostal spaces. Measurements taken included the eye muscle area, meat color, shear force (after 24 h of cold storage), centrifugal moisture loss, and drip loss. Portions of the meat samples were sealed in bags and stored at −20°C for subsequent analysis of meat nutritional indicators.

### 2.2 Apparent data collection

Throughout the main trial, at a 5-day interval and specifically at 8:30 a.m., feed remnants from each group were collected, weighed, and meticulously recorded. These remnants were then subjected to a 48-h drying process in a 65°C oven, after which they were reweighed to determine their final weight. Consumption was calculated as the difference between the total feed quantity provided the previous day and the weight of the remaining feed the following day. Furthermore, on days 1, 45, and 90 of the trial, prior to morning feeding, cattle were individually weighed using electronic scales to ensure precision within a margin of error of less than 0.5 kg, and these measurements were accurately recorded for analysis.

### 2.3 Nutritional composition of feed and meat quality

The nutrient composition of the feed, including crude protein (CP), dry matter (DM), and ether extract (EE), was determined using the methods of the Association of Official Analytical Chemists (AOAC, 1990). The acid detergent fiber (ADF) and neutral detergent fiber (NDF) content in the feed were analyzed using the method described by Van Soest et al. (1991), with a fiber analysis instrument. Starch, calcium (Ca), and phosphorus (P) levels were assessed using near-infrared spectroscopy (NIRS). The net energy content of the feed was calculated using the methodology outlined in the “Institute of Animal Husbandry et al. (2004).” For meat quality assessment, the eye muscle area was outlined on a grid sulfuric acid paper between the 12th and 13th rib cuts, and then, the area was calculated. After slaughter, meat samples were stored under low light conditions for 45 min, followed by color analysis using a calibrated colorimeter (NR10QC, 3nh Company). Five distinct points on each sample were selected for color measurement, and the average value was recorded. Shear force was measured by taking meat samples with a core sampler, sealing them in bags, and heating in a 75°C water bath until the center of the meat reached 70°C. Following cooling, surface moisture was blotted dry with filter paper. Meat strips, 1 × 1 cm in cross-section, were cut perpendicular to the muscle fibers using a texture analyzer (tenderness meter). For each meat sample, 10 measurements were taken and the average was calculated. Drip loss and centrifuged water loss in meat samples were evaluated following the 2015 “Objective Evaluation Method for Meat Quality” from the Agricultural Industry Standards of the People’s Republic of China. Frozen meat samples were pulverized and then sieved through an 18-mesh screen. The CP and EE content in meat samples were analyzed using AOAC (1990) methods. The analysis of amino acid and fatty acid profiles in the meat samples was conducted by Shanghai Tianxiang Quality Technology Service Co., Ltd.

### 2.4 Rumen fermentation parameters

For the analysis of the rumen fluid, volatile fatty acid (VFA) concentrations were determined using gas chromatography, following the method described by Erwin et al. (1961), with an Agilent 6890B gas chromatograph (Leiden Scientific Instruments Co., Ltd., Suzhou City, Jiangsu Province, China) equipped with an HP-INNOWAX capillary column (30.0 m × 320 μm × 0.5 μm, Catalog No: 19091 N-213). Ammonia nitrogen (NH_3_-N) concentration in the rumen fluid was measured using a colorimetric method employing a Biotron Synergy H1 enzyme-linked analyzer (Guangzhou Huqiao Instrument Technology Co., Ltd., Guangzhou City, Guangdong Province, China).

### 2.5 Rumen fluid 16S DNA sequencing and analysis

Genomic DNA was extracted from rumen fluid samples using magnetic bead-based extraction, followed by an evaluation of DNA purity and concentration. Upon confirming that the standards were met, the samples were diluted with sterile water to achieve a concentration of 1 ng/μl. Primers specific to the 16S V3-V4 region (515F and 806R) were used for amplification. The PCR mixture, consisting of 15 μL Phusion® High-Fidelity PCR Master Mix (New England Biolabs), 0.2 μM primers, and 10 ng genomic DNA template, was subjected to an initial denaturation at 98°C for 1 min, 30 cycles of denaturation at 98°C for 10 s, annealing at 50°C for 30 s, extension at 72°C for 30 s, and a final extension at 72°C for 5 min. Gel electrophoresis was used to analyze the PCR products, followed by enzymatic quantification of the purified products. After quantification, samples were pooled in equimolar ratios and were subjected to electrophoresis for the confirmation of target band presence. Library construction was carried out using the NEB Next® Ultra™ II FS DNA PCR-free Library Prep Kit (NEB/E7430L). After library preparation, quantification was conducted using Qubit and quantitative PCR (Q-PCR) methods. Once qualified, the libraries underwent paired-end (PE) 250 sequencing on a NovaSeq 6,000 platform. Following sequencing, sample data were demultiplexed based on barcodes and PCR primer sequences. Barcodes and primer sequences were removed using FLASH software (Version 1.2.11) (Magoč and Salzberg, 2011). Raw tags for each sample were then assembled into original tag data (raw tags). Raw tags were subjected to rigorous filtering and quality control using fastp software (Version 0.23.1) (Bokulich et al., 2013), resulting in high-quality tag data (clean tags). Following processing, clean tags were further analyzed to eliminate chimeric sequences. Tag sequences were aligned with species annotation databases (Silva for 16S/18S, Unite for ITS) to detect and remove chimeric sequences, yielding the final effective data (effective tags) (Edgar et al., 2011). Effective tags underwent denoising, employing the DADA2 module or deblur within QIIME2 software (version QIIME2-202006, defaulting to DADA2), to obtain final amplicon sequence variants (ASVs) and a feature table (Wang et al., 2021). Taxonomic annotation of ASVs was performed using QIIME2 software and Silva databases 138.1 for 16S/18S and Unite v8.2 for ITS. Rapid multiple sequence alignment of all ASV sequences was conducted using QIIME2 software for phylogenetic tree construction. Data from all samples were normalized based on the sample with the lowest data volume, followed by alpha and beta diversity analyses using the normalized data.

### 2.6 Data processing and statistical analysis

Experimental data were initially processed and formatted using Microsoft Excel. The statistical analysis was performed using the one-way analysis of variance (ANOVA) in SPSS 2.0, with post-hoc comparisons conducted via LSD and Duncan’s multiple range tests. The *p*-value was used to determine statistical significance among the three groups. Experimental data are presented in tables as mean ± standard error of the mean (SEM), with significance denoted by *p* < 0.05.

## 3 Results

### 3.1 Growth performance

**Figure 1:**
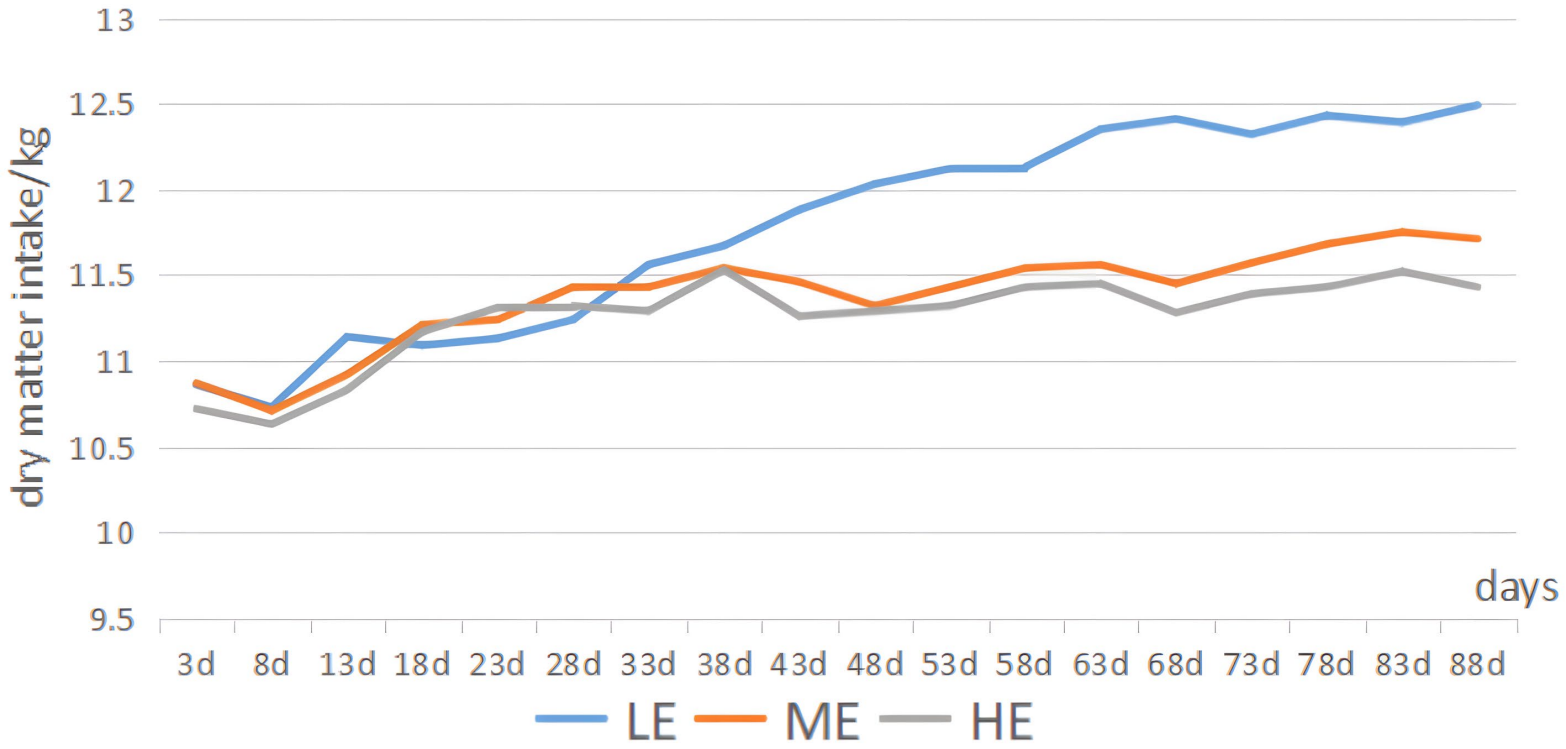
Trend in DMI variations.

Figure 1 illustrates that, during the experimental period, the HE group showed a 1.1% decrease in the average daily matter intake (ADMI) compared to the ME group (*p* < 0.05) and a 4.4% decrease compared to the LE group (*p* < 0.05). The ME group exhibited a 3.4% decrease in ADMI relative to the LE group (*p* < 0.05). Notably, ADMI in all groups demonstrated an increasing trend throughout the experimental period. Table 2 reveals that, in terms of overall daily weight gain (ADG), the HE group showed a 25.6% increase compared to the LE group (*p* < 0.05), and the ME group exhibited a 30.5% increase (*p* < 0.05). Regarding the overall feed-to-weight ratio, the LE, ME, and HE groups recorded ratios of 14.78, 11.32, and 11.17, respectively. The HE and ME groups exhibited reductions in feed-to-weight ratio of 24.4 and 23.4%, respectively, compared to the LE group. In the 0–45 day period, the feed-to-weight ratios for the LE, ME, and HE groups were 15.92, 13.35, and 10.30, respectively. The HE and ME groups showed reductions in the feed-to-weight ratio of 35.3 and 16.1%, respectively, compared to the LE group. During the 45–90 day period, the feed-to-weight ratios for the LE, ME, and HE groups were 14.78, 10.42, and 13.24, respectively. The HE and ME groups exhibited feed-to-weight ratio reductions of 10.4 and 29.5%, respectively, compared to the LE group.

**Table 2:** Effects of dietary energy level on Angus steer during the late fattening period.

Items		Group			^1^SEM	^2^P
		LE group	ME group	HE group		
Initial weight/kg		521.40	521.60	522.60	4.87	1.00
Body weight on the 45th day/kg		556.40	563.60	575.00	5.59	0.41
Body weight on the 90th day/kg		594.90	614.30	618.60	5.71	0.20
0–45 Days	ADG/kg	0.78^b^	0.93^ab^	1.16^a^	0.06	0.03
45–90 Days	ADG/kg	0.86^b^	1.15^a^	0.97^ab^	0.05	0.03
Whole period	ADG/kg	0.82^b^	1.03^a^	1.07^a^	0.04	0.02

^1^SEM values represent the mixed standard errors between groups, as indicated in the following table. ^2^*p* indicate the significance among the three groups, as shown in the table. ^a,b^Values within a row with no common superscripts differ significantly.

### 3.2 Meat quality and nutritional composition of meat

Across different experimental groups with varying dietary energy levels, there were no significant differences in parameters such as the eye muscle area, shear force, centrifugal dehydration rate, drip loss rate, meat color, and crude protein. However, the Longissimus dorsi muscle of the HE group exhibited a significantly higher crude fat content compared to the ME (*p* < 0.05) and the LE groups (*p* < 0.05). According to the analysis results, dietary energy levels had no significant impact on sensory evaluation indicators of beef, as shown in Table 3. Table 4 shows that varying dietary energy levels did not significantly impact the amino acid levels in the Longissimus dorsi muscle of Angus steers. However, dietary energy levels significantly affected the total fatty acid content in the muscle. The muscle of the HE group had a significantly higher total fatty acid content compared to both the ME and LE groups (*p* < 0.05). For unsaturated fatty acids, the HE group’s linoleic acid (octadecadienoic acid) content was significantly higher than in the ME and LE groups (*p* < 0.05). Additionally, pentadecanoic acid and arachidonic acid were exclusively found in the muscle of the HE group.

**Table 3:** Effects of dietary energy levels on the meat quality of late-fattening Angus steers.

Items	LE	ME	HE	SEM	P
Nutritional quality of meat					
CP, %	21.59	22.13	21.39	0.46	0.19
Crude Fat, %	7.89^b^	8.38^b^	9.17^a^	0.5	0.01
Physical quality					
Eye Muscle Area, cm^2^	153.43	160.3	159.89	7.18	0.97
Shear Force, N	178.42	181.31	168.32	5.43	0.76
Centrifugal Dewatering Rate, %	6.03	5.79	5.73	0.33	0.8
Drip Loss Rate, %	11.4	10.98	11.59	0.77	0.2
Meat color					
L*	31.34	32.28	30.98	0.63	0.81
a*	14.34	15.12	13.98	0.39	0.62
b*	4.32	4.17	4.76	0.24	0.33

^a,b^Values within a row with no common superscripts differ significantly.

**Table 4:** Effects of daily energy intake levels on the nutritional composition of Angus steer beef during the late fattening period.

Items	LE	ME	HE	SEM	P
Aspartic acid^$^	1.47	1.40	1.38	0.03	0.60
Phenylalanine^$&£^	0.68	0.64	0.64	0.02	0.61
Alanine^$^	0.94	0.88	0.89	0.02	0.52
Methionine^£^	0.44	0.42	0.42	0.01	0.68
Proline	0.69	0.63	0.65	0.02	0.44
Glycine^$^	0.76	0.70	0.70	0.02	0.54
Glutamic acid^$^	2.23	2.13	2.10	0.06	0.68
Arginine	1.00	0.97	0.98	0.02	0.88
Lysine^£^	1.47	1.40	1.40	0.03	0.70
Tyrosine^$^	0.58	0.57	0.57	0.01	0.96
Leucine^£^	1.30	1.30	1.28	0.03	0.94
Serine	0.60	0.57	0.57	0.01	0.77
Threonine^£^	0.75	0.72	0.72	0.02	0.70
Valine^£^	0.84	0.71	0.72	0.03	0.25
Isoleucine^£^	0.74	0.69	0.70	0.02	0.56
Histidine	0.69	0.67	0.68	0.02	0.91
Total amino acids	15.13	14.43	14.38	0.36	0.69
Tetradecanoic acid	0.19	0.19	0.32	0.03	0.07
Pentadecanoic acid	<0.01	<0.01	0.03	0.01	0.19
Hexadecanoic acid	1.85	1.94	3.33	0.31	0.05
Heptadecanoic acid	0.03	0.03	0.10	0.02	0.05
Octadecanoic acid	0.97	0.94	1.71	0.16	0.06
Tetradecenoic acid	0.03	0.04	0.09	0.01	0.17
Hexadecenoic acid	0.23	0.27	0.47	0.05	0.11
Octadecenoic acid	2.27	2.33	4.32	0.45	0.06
Octadecadienoic acid	0.13^b^	0.14^b^	0.21^a^	0.02	0.03
Arachidonic acid	0.00	0.00	0.03	0.01	0.19
Total fatty acids	5.71^b^	5.88^b^	13.10^a^	1.41	0.01

^£^Essential Amino acids; ^$^Savory Amino acids. ^a,b^Values within a row with no common superscripts differ significantly.

### 3.3 Rumen fermentation parameters

Table 5 shows the fermentation parameters of the rumen fluid for each experimental group. Dietary energy levels were observed to have no significant effect on the rumen fluid pH and NH_3_-N content but significantly impacted various volatile fatty acids. In the LE group, the concentration of acetic acid in the rumen fluid was significantly higher compared to the ME group (*p* < 0.05) and the HE group (*p* < 0.05). The concentration of propionic acid in the HE group’s rumen fluid was significantly higher than in both the LE and ME groups (*p* < 0.05). Valeric acid concentration in the HE group was significantly higher than in the ME group (*p* < 0.05), and isovaleric acid concentration was notably higher compared to both the LE and ME groups (*p* < 0.05). The total volatile fatty acid concentration was significantly higher in the HE group compared to the ME group (*p* < 0.05). Regarding the acetic acid/propionic acid ratio, the LE group exhibited significantly higher values than both the ME and HE groups (*p* < 0.05). As dietary energy levels increased, there was a decrease in the molar ratio of acetic acid (*p* < 0.01) and an increase in the molar ratio of propionic acid (*p* < 0.01).

**Table 5:** Effects of dietary energy levels on rumen fermentation parameters in late-stage fattening Angus steers.

Items	LE	ME	HE	SEM	P
PH	6.96	6.93	6.79	0.65	0.01
NH_3_-N	8	8.17	8.21	0.59	0.13
Acetic acid, ng/μL	33.36^a^	22.03^b^	25.81^b^	1.64	0.01
Propionic acid, ng/μL	7.83^b^	6.90^b^	10.40^a^	0.54	0.02
Butyric acid, ng/μL	7.06	3.27	6.15	0.06	0.66
Isobutyric acid, ng/μL	1.2033	1.3244	1.21	0.64	0.04
Valeric acid, ng/μL	0.33^ab^	0.19^b^	0.62^a^	0.10	0.00
Isovaleric acid, ng/μL	1.27^b^	1.35^b^	2.02^a^	0.07	0.03
Total volatile fatty acids, ng/μL	51.05^ab^	35.06^a^	46.21^b^	2.73	0.04
Acetic acid to propionic acid ratio	4.38^A^	3.30^B^	2.52^C^	0.18	<0.01
Molar ratios, %					
Acetic acid	75.27^A^	73.86^AB^	67.83^C^	0.83	<0.01
Propionic acid	14.13^C^	18.68^B^	22.11^A^	0.79	<0.01
Butyric acid	10.60^a^	7.47^b^	10.07^a^	0.50	0.02

^a,b^Values within a row with no common superscripts differ significantly. ^A–C^No common superscript uppercase letters indicate highly significant differences.

### 3.4 16S rRNA sequencing and annotation analysis

**Figure 2:**
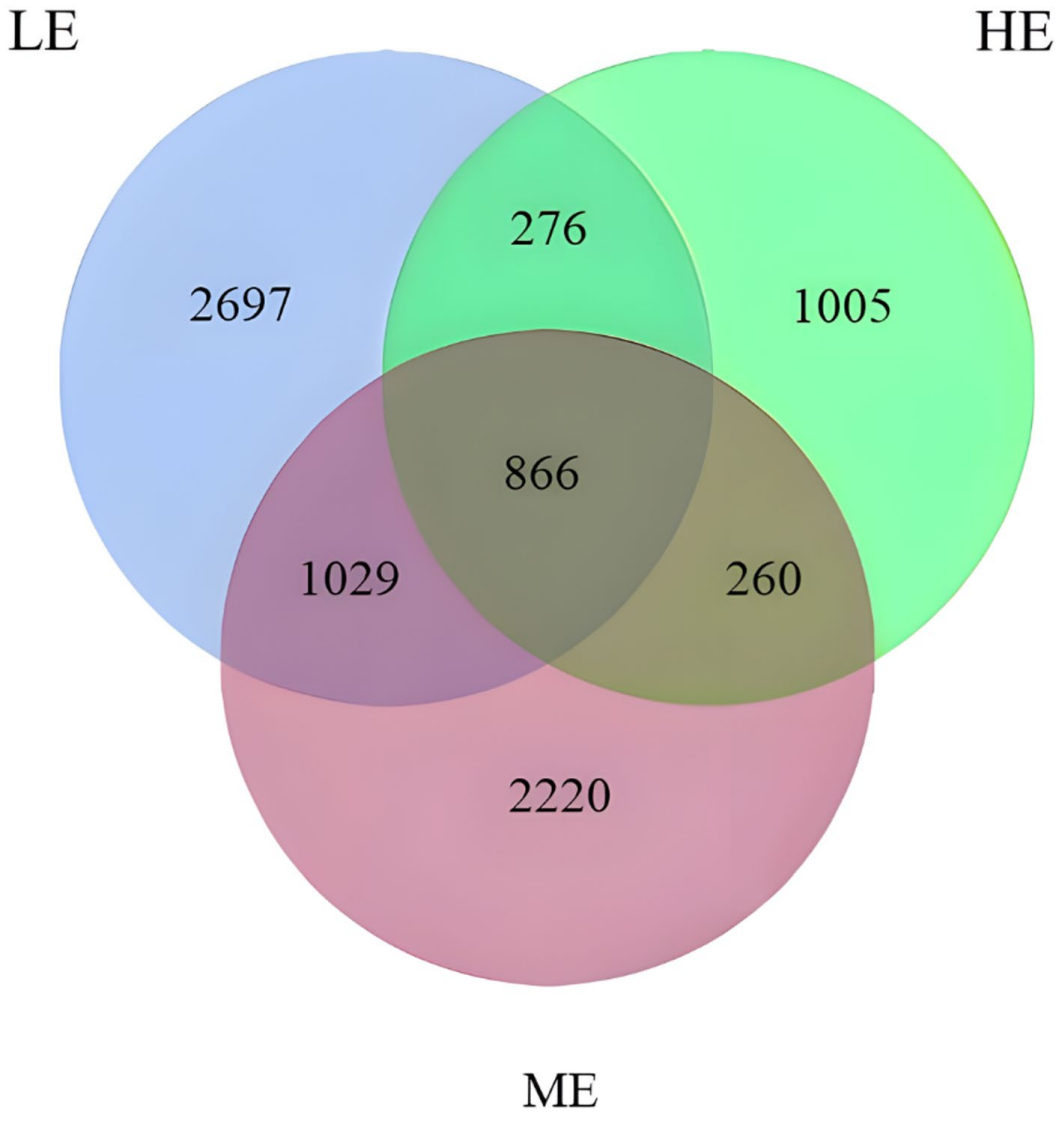
Venn diagram of OTU statistics of rumen bacteria.

According to Figure 2, sequencing of the V3 and V4 regions in this experiment resulted in 2,091,965 raw data reads, with 1,747,504 high-quality sequences obtained after quality control. The sequences were clustered into 8,353 operational taxonomic units (OTUs) using a 97% similarity threshold. The clustering diagram revealed that the LE group possessed the highest number of unique OTUs, indicating a more diverse rumen microbiota compared to the ME and HE groups. Notably, the HE group demonstrated the lowest diversity in rumen microbiota.

### 3.5 Microbial diversity in the ruminal fluid

Table 6 indicates that the LE group displayed significantly higher values in the chao1, observed_features, Shannon, and Simpson metrics compared to the HE group (*p* < 0.01). Additionally, the LE group exceeded the ME group in chao1, observed_features, and Shannon metrics (*p* < 0.01). Conversely, the ME group outperformed the HE group in all four metrics, although these differences were not statistically significant. Therefore, it is evident that the LE group possesses the highest diversity in rumen fluid microbiota.

**Table 6:** Comparison of alpha diversity in rumen fluid microbiota.

	LE	ME	HE	SEM	P
chao1	1061.62^A^	719.55^B^	466.22^B^	78.34	0.004
observed_features	1054.78^A^	713.11^B^	462.89^B^	77.65	0.004
Shannon	7.04^A^	4.91^B^	3.67^B^	0.47	0.008
Simpson	0.87^a^	0.69^ab^	0.63^b^	0.04	0.030

^a,b^Values within a row with no common superscripts differ significantly. ^A,B^No common superscript uppercase letters indicate highly significant differences.

**Figure 3:**
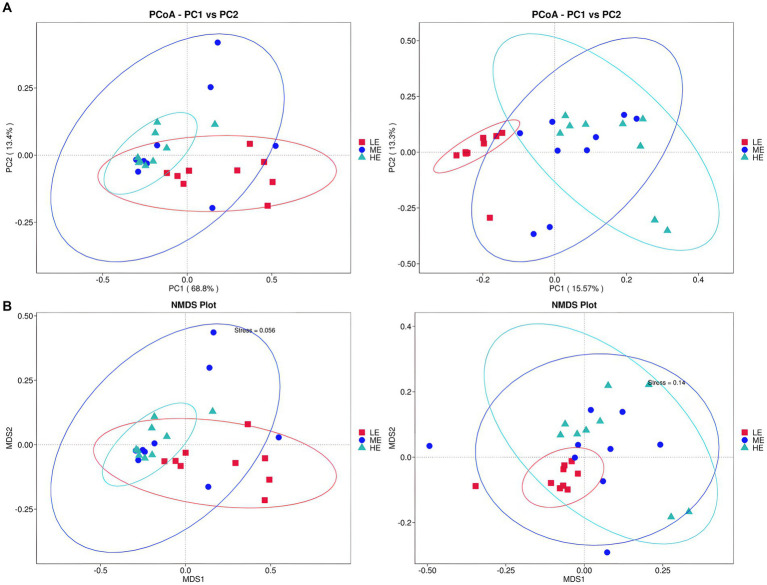
Alpha diversity analysis of rumen flora. **(A)** Two-dimensional PCoA plot of rumen fluid microbiota. **(B)** NMIDS plot of rumen fluid microbiota. Left: based on weighted UniFrac distance; Right: based on unweighted UniFrac distance.

In Figure 3, the ME and HE groups demonstrate similar distribution patterns, characterized by closely clustered samples within each group. The LE group, while showing some overlap with the ME and HE groups, predominantly occupies a distinct region. This suggests that increased dietary energy levels have, to some extent, altered the structure of the rumen microbiota. According to Tables 7, 8, a smaller Observe-Delta value signifies less variation within groups, whereas a larger Expect-Delta value indicates more pronounced differences between groups. A positive A value signifies that differences between groups exceed those within groups, while a negative A value suggests the opposite, with greater differences within groups than between groups. It is evident that significant differences exist between the LE group and both the ME and HE groups, while the differences between the ME and HE groups are not significant. This finding corroborates the previous observation of higher rumen microbiota diversity in the LE group.

**Table 7:** MRPP analysis of inter-group differences.

Group	A	Observed-delta	Expected-delta	Significance
LE-ME	0.05175	0.64976	0.68522	0.025
LE-HE	0.13108	0.5229	0.60178	0.001
ME-HE	0.01135	0.53437	0.54051	0.193

**Table 8:** Adonis analysis of inter-group differences.

Group	Df	Sums of Sqs	Mean Sqs	F. Model	R^2^	Pr (>F)
LE-ME	1 (16)	0.5853 (3.86617)	0.5853 (0.24164)	2.42222	0.1315 (0.86852)	0.027
LE-HE	1 (16)	1.0048 (2.48229)	1.0048 (0.15514)	6.47677	0.2882 (0.71185)	0.001
ME-HE	1 (16)	0.2739 (2.87614)	0.2739 (0.17976)	1.52394	0.0870 (0.91304)	0.136

### 3.6 Composition of the rumen bacterial community

The statistical analysis identified the top 20 bacterial relative abundances at both the phylum and genus levels. Comparisons were conducted among the different experimental groups to identify differences. Table 9 shows that the relative abundance of Proteobacteria in the HE group is significantly higher than in the LE group (*p* < 0.05) but not significantly different from the ME group. Relative abundances of Bacteroidota (*p* < 0.05), Spirochaetota (*p* < 0.05), and Fibrobacterota (*p* < 0.01) are significantly higher in the LE group than in the HE group. Table 10 indicates that the relative abundance of the *Succinivibrio* genus in the HE group is significantly higher than in both the LE and ME groups (*p* < 0.05). In the LE group, bacterial relative abundances of the *Prevotella* and *Ruminococcus* genera were significantly higher than in the HE group (*p* < 0.01). Bacterial relative abundances of the UCG-005, CAG-352, UCG-002, UCG-010, and *Kroppenstedtia* genera were all significantly higher in comparison to those in the HE group (*p* < 0.05).

**Table 9:** Effects of dietary energy levels on the relative abundance (%) of rumen bacterial genera.

	LE	ME	HE	SEM	P
Proteobacteria	29.78^b^	48.89^ab^	63.93^a^	27.11	0.02
Firmicutes	35.97	35.63	30.94	13.44	0.69
Bacteroidota	31.96^a^	12.32^b^	4.23^b^	18.53	<0.01
Actinobacteriota	0.64	0.81	0.53	0.66	0.67
Acidobacteriota	0.24	0.47	0.00	0.90	0.55
Patescibacteria	0.20	0.48	0.02	0.47	0.12
Spirochaetota	0.27^a^	0.12^ab^	0.03^b^	0.19	0.02
Fusobacteriota	0.04	0.19	0.16	0.28	0.50
Desulfobacterota	0.08	0.18	0.05	0.28	0.63
Nitrospirota	0.08	0.17	0.00	0.33	0.55
Euryarchaeota	0.16	0.07	0.02	0.14	0.07
Fibrobacterota	0.19^A^	0.01^B^	0.01^B^	0.14	<0.01
Chloroflexi	0.06	0.11	0.00	0.22	0.55
Gemmatimonadota	0.04	0.09	0.00	0.17	0.55
Campilobacterota	0.00	0.06	0.01	0.07	0.09
Myxococcota	0.02	0.05	0.00	0.10	0.50
MBNT15	0.02	0.04	0.00	0.08	0.57
Verrucomicrobiota	0.01	0.04	0.01	0.03	0.19
Cyanobacteria	0.01	0.03	0.01	0.03	0.40
Elusimicrobiota	0.02	0.02	0.01	0.02	0.52

^a,b^Values within a row with no common superscripts differ significantly. ^A,B^No common superscript uppercase letters indicate highly significant differences.

**Table 10:** Effects of dietary energy levels on the relative abundance (%) of rumen bacterial genera.

	LE	ME	HE	SEM	P
Pseudomonas	27.20	44.16	58.80	5.43	0.05
Eubacterium_coprostanoligenes_group	7.39	5.67	4.08	0.76	0.21
Rikenellaceae_RC9_gut_group	1.10	7.41	6.70	1.16	0.05
Prevotella	9.74^A^	2.20^B^	0.31^B^	1.27	<0.01
NK4A214_group	5.21	4.05	1.93	0.79	0.23
Muribaculaceae	2.20	3.88	4.05	0.81	0.61
Ruminococcus	7.09^A^	2.23^B^	0.57^B^	0.85	<0.01
Clostridia_UCG-014	2.03	3.45	2.20	0.55	0.53
F082	2.97	1.66	1.28	0.38	0.16
UCG-005	3.66^a^	1.15^b^	0.12^b^	0.55	0.02
Bacillus	1.68	1.22	1.45	0.23	0.74
CAG-352	2.16^a^	1.15^b^	0.61^b^	0.21	0.01
Succinivibrio	0.57^b^	0.57^b^	1.82^a^	0.23	0.04
Oceanobacillus	0.54	0.87	1.36	0.32	0.58
UCG-002	1.59^a^	0.66^b^	0.47^b^	0.19	0.03
Virgibacillus	0.53	0.60	1.53	0.23	0.13
Christensenellaceae_R-7_group	1.47^a^	0.53^b^	0.38^b^	0.17	0.01
UCG-010	1.43^a^	0.70^ab^	0.21^b^	0.19	0.02
Saccharofermentans	0.93	0.74	0.40	0.12	0.16
Kroppenstedtia	0.77^a^	0.74^a^	0.28^b^	0.09	0.03

^a,b^Values within a row with no common superscripts differ significantly. ^A,B^No common superscript uppercase letters indicate highly significant differences.

**Figure 4:**
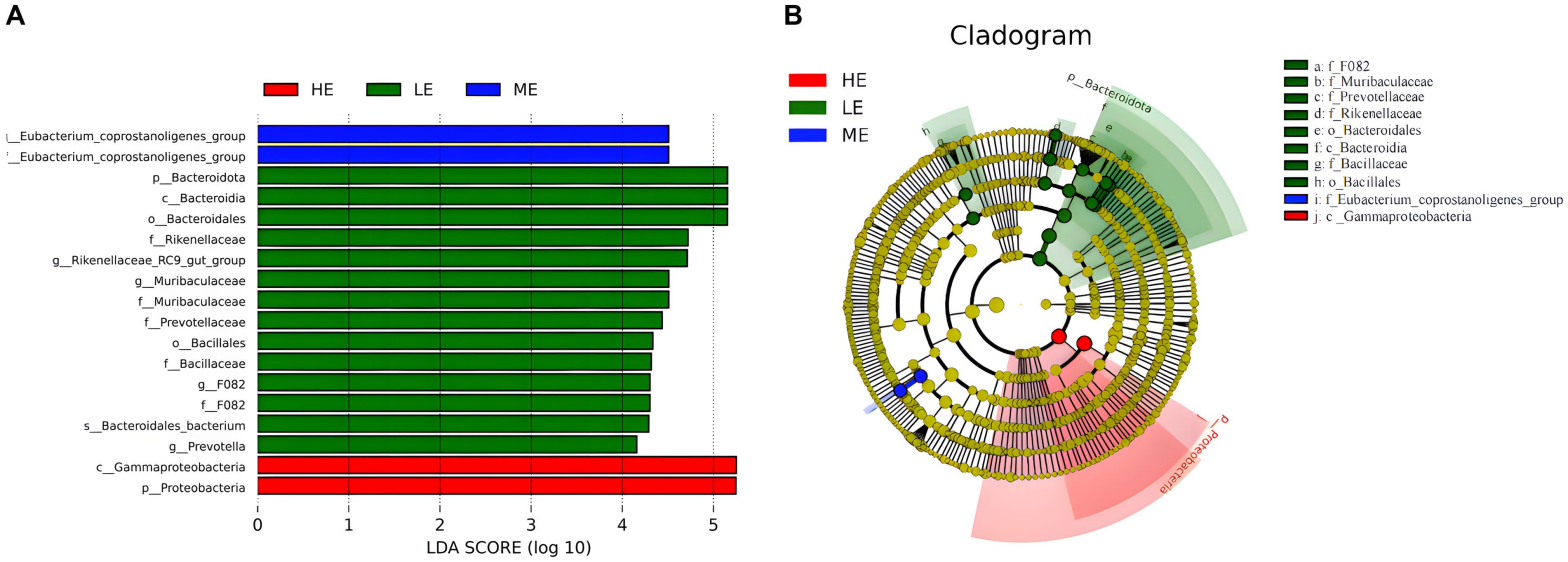
LEfSe analysis of rumen microbiota. **(A)** LDA bar chart, **(B)** LEfSe evolution branch diagram. The graph reflects the affiliation of flora populations between groups from the species to phyla levels, with node size corresponding to the average relative abundance of the corresponding taxon.

Figure 4A reveals 18 distinct taxonomies among the experimental groups, encompassing 1 species, 5 genera, 6 families, 2 orders, 2 classes, and 2 phyla. Of these, 14 taxonomies were found in the LE group, while 2 were identified in each of the ME and HE groups. At the phylum level, a significant increase was observed in the relative abundance of Proteobacteria in the HE group and Bacteroidota in the LE group (*p* < 0.05). At the genus level, the LE group showed a significant increase in the abundance of Eubacterium_coprostanoligenes_group, Prevotella, and Muribaculaceae and a notable decrease in Rikenellaceae_RC9_gut_group (*p* < 0.05). In the evolutionary tree, the LE, ME, and HE groups exhibit distinct evolutionary pathways, indicating that varying dietary energy levels lead to different evolutionary directions in the rumen microbiota (Figure 4B).

### 3.7 Correlations between rumen microbiota and rumen fermentation parameters

**Figure 5:**
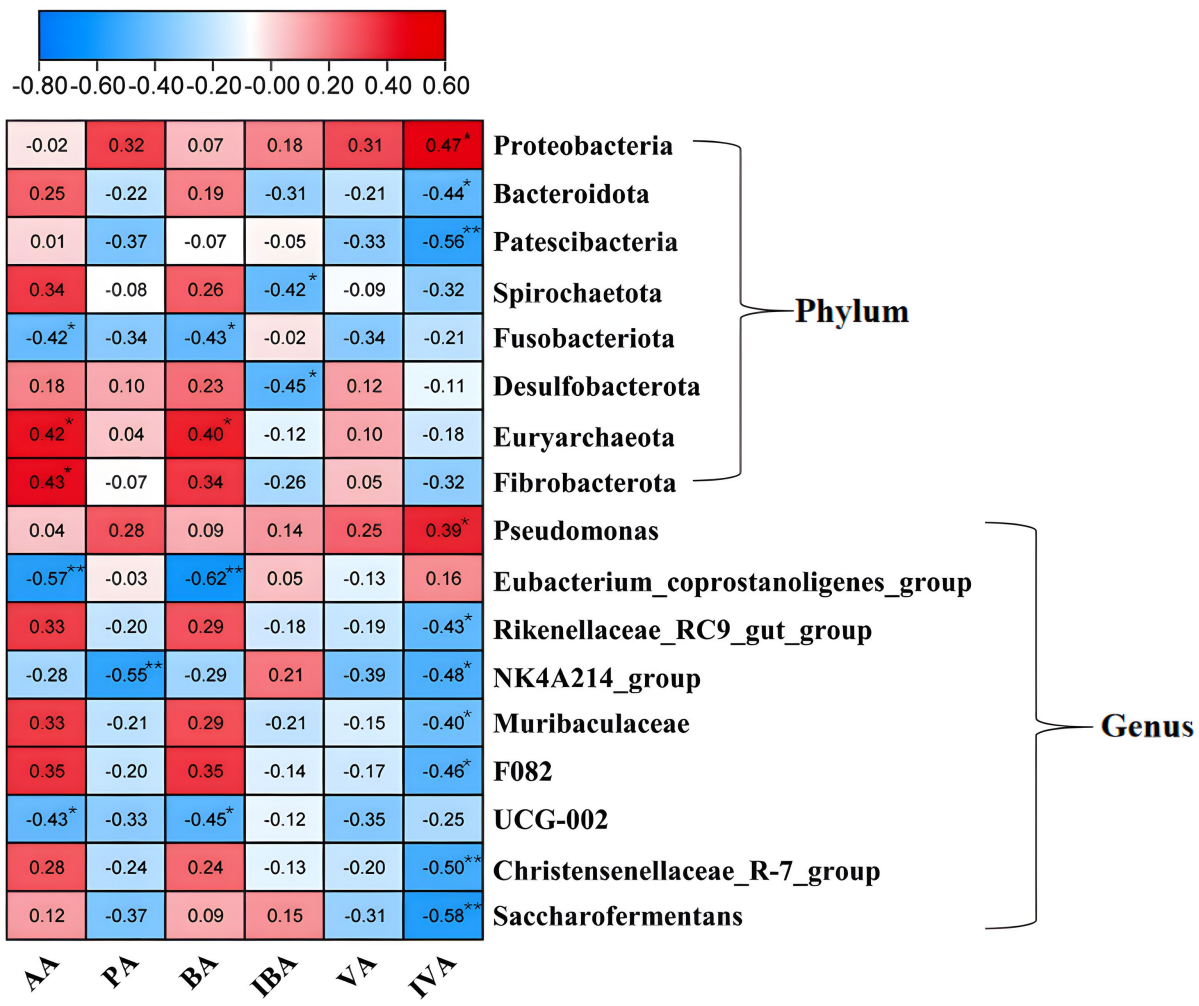
Correlation of fermentation parameters and flora. *Indicates *p* < 0.05 and **indicates *p* < 0.01.

Pearson correlation analysis was performed to analyze the correlation between fermentation parameter rates (FPR) and rumen microbiota, revealing associations between eight phyla and nine genera with rumen fermentation parameters (Figure 5). At the phylum level, Proteobacteria showed a positive correlation with isovaleric acid (*p* < 0.05), whereas Bacteroidota and Patescibacteria both exhibited negative correlations with isovaleric acid (*p* < 0.05). Spirochaetota and Desulfobacterota were negatively correlated with isobutyric acid (*p* < 0.05). Euryarchaeota was positively correlated with acetic acid and butyric acid (*p* < 0.05), while Fusobacteria negatively correlated with these acids (*p* < 0.05). Fibrobacterota showed a positive correlation with acetic acid (*p* < 0 0.05). At the genus level, *Pseudomonas* was positively correlated with isovaleric acid (*p* < 0.05). Conversely, the Eubacterium_coprostanoligenes_group and UCG-002 genera were negatively correlated with acetic acid and butyric acid (*p* < 0.01 for Eubacterium_coprostanoligenes_group; *p* < 0.05 for UCG-002). Similarly, Rikenellaceae_RC9_gut_group, Christensenellaceae_R-7_group, Saccharofermentans, Muribaculaceae, and F082 genera were all negatively correlated with isovaleric acid (*p* < 0.05 for Rikenellaceae_RC9_gut_group; *p* < 0.01 for others). The NK4A214_group was negatively correlated with both propionic acid (*p* < 0.01) and isovaleric acid (*p* < 0.05).

## 4 Discussion

Feed energy level is a primary influencing factor in animal production. This results of this study indicate that a high-energy diet contributes to improved average daily weight gain and feed-to-weight ratio. These findings are similar to the research by Wang et al. (2019). In the first 45 days, the HE group demonstrated a more favorable feed-to-weight ratio than the ME group, whereas from 45 to 90 days, the ME group showed a better feed-to-weight ratio than the HE group. This can be attributed to the higher energy level, starch, and lipid content in the diet of the HE group compared to the ME and LE groups, impacting rumen fermentation in beef cattle and reducing nutrient absorption efficiency. Throughout the trial, ADMI in each experimental group decreased as the dietary energy level increased. This suggests that, at the dietary energy levels used in the experimental groups, gastrointestinal filling is not the main determinant of feed consumption, aligning with the findings of Krehbiel (Krehbiel, 2022).

The composition of various fatty acids in meat is crucial in determining its quality, influencing both flavor and the health implications of dietary choices (Wood et al., 2004). In this study, the ratio of polyunsaturated to saturated fatty acids in the Longissimus dorsi muscle of the LE, ME, and HE groups exceeded the standard of 0.4, indicating a beneficial fat intake source. Observations revealed that the HE group possessed significantly higher fatty acid contents compared to the ME and LE groups. Significantly, pentadecanoic acid and arachidonic acid (C20:4) were detected exclusively in the muscle of the HE group. The observed phenomenon is likely attributable to the increased levels of crude fat and bypass fatty acid in the diet of the HE group, which provide direct precursors for the synthesis of pentadecanoic acid and arachidonic acid. Upon examining the changes in the rumen microbiota, a significant increase in the abundance of Proteobacteria within the HE group was noted. As a class of bacteria known to metabolize unsaturated fatty acids (Fujita et al., 2007), their activity could have facilitated the synthesis or transformation of arachidonic acid. This could potentially explain the increase in arachidonic acid levels that were detected exclusively in the cattle of the HE group. This suggests that increased dietary energy levels can significantly augment the fatty acid content in the Longissimus dorsi muscle of Angus steers. Additionally, as dietary energy levels increase, there is a notable rise in the ratio of unsaturated to saturated fatty acids along with a significant increase in the unsaturated fatty acid content, potentially rendering beef, a healthier source of lipids. However, this might be related to the inclusion of rumen bypass fat in the diet.

Ruminant animals’ rumen environment is regulated by various factors and exhibits a strong capacity for maintaining stability and self-regulation, as corroborated by several studies (Wittek et al., 2018). NH_3_-N is a product of protein and non-protein nitrogen metabolism in the rumen. The concentration of NH_3_-N is dependent on dietary crude protein levels and the rate of microbial breakdown and metabolism. Higher concentrations of NH_3_-N generally favor microbial protein synthesis (Basso et al., 2018; Lara et al., 2018). In this study, no significant differences in rumen fluid NH_3_-N concentrations were observed among the groups. Despite varying dietary energy levels, the rumen’s self-regulation capability ensured relatively stable rates of protein and non-protein nitrogen metabolism. Research indicates that ruminal microbes can convert ammonia nitrogen into microbial protein, a process that is directly influenced by the supply of fermentable carbohydrates in the diet (Zurak et al., 2023). In this study, the consistent provision of crude protein across diets with varying energy levels ensured the continuous utilization of nitrogen sources by ruminal microbes, thereby aiding in the maintenance of stable ammonia nitrogen (NH_3_-N) concentrations within the rumen. An increase in the dietary starch content facilitated the growth and fermentative activities of ruminal microbes, thereby enhancing nitrogen utilization efficiency and reducing ammonia accumulation. Conversely, reducing the fiber content helped moderate the rate of ruminal fermentation, further decreasing the production of excess ammonia. These mechanisms collaboratively contribute to the stabilization of NH_3_-N concentrations within the rumen.

Acetic acid serves as a primary precursor for fat synthesis, whereas propionic acid mainly contributes to glucose synthesis. Consequently, higher levels of propionic acid are associated with improved animal production performance (Kristensen and Harmon, 2004). This experiment observed that, with increased feed energy levels, the concentration of total VFAs in the rumen initially decreased and then increased. Furthermore, the VFA concentration in both the ME and HE groups was notably lower than in the LE group. This reduction in VFA concentration may be attributed to the higher carbohydrate content in high-energy feeds. However, the increased energy intake correlated with an enhanced daily weight gain. With increasing dietary energy levels, acetic acid concentration decreased, whereas propionic acid concentration increased. This suggests a significant shift toward propionic acid fermentation with higher dietary energy levels, as indicated by the increased proportion of propionic acid in VFAs. Several studies have shown that high-energy diets decrease the proportion of acetic acid, as acetic acid is predominantly produced through non-carbohydrate fermentation. An increase in dietary energy levels typically leads to a reduced proportion of NDF and acid ADF, increased soluble carbohydrates, and higher propionic acid concentration from rumen fermentation. Propionic acid, which is vital for glucose synthesis in the body, implies that an increase in its content enhances energy availability of the body for growth (Askar et al., 2023).

Branched chain fatty acids and valeric acid (isoacids) are crucial for the growth of many rumen microorganisms and indispensable for cellulose digestion (Muller, 1987). The correlation analysis reveals a positive association between Proteobacteria, Pseudomonas, and isovaleric acid in the rumen microbial community. The abundances of Proteobacteria and Pseudomonas in the HE group are significantly higher than in the LE and ME groups. The isovaleric acid concentration in the HE group is significantly higher than in the ME and LE groups. This suggests that a high-energy diet enhances cellulose digestion. This may be one of the contributing factors to the daily weight gain in the HE and ME groups, being significantly higher than that in the LE group.

In this study, the HE group displayed fewer OTUs compared to the LE and ME groups. This finding suggests that a high-energy diet may lead to reduced diversity in rumen microbiota. Significant differences in microbial distribution were observed in the LE group compared to the ME and HE groups. This indicates that variations in dietary energy levels can influence the species distribution within rumen microbiota. Research has shown that rumen microbiota predominantly consist of Bacteroidetes and Firmicutes. The sequencing results of this experiment corroborate this observation (Ley et al., 2008; Singh et al., 2012). This study found that, compared to the LE group, the abundance of Proteobacteria significantly increased, while the abundance of Bacteroidota significantly decreased in the HE group. This phenomenon may be linked to the increased proportion of corn and the reduced inclusion of corn stover silage in the HE group’s feed, leading to a decrease in the fiber content and an increase in easily fermentable carbohydrates in the feed. Such changes in feed composition may have promoted the increase in the abundance of Proteobacteria, which utilize easily fermentable carbon sources, resulting in a decrease in the abundance of fiber-degrading bacteria, Bacteroidota. This finding is in line with the research conducted by Fernando et al. (2010), who observed that an increase in the proportion of concentrate in the feed leads to an increase in the abundance of Proteobacteria and a decrease in the abundance of Bacteroidetes. It was observed that, with an increase in dietary energy levels, the abundance of Bacteroidetes decreased, while Firmicutes remained consistent across groups. The study by Ley revealed a lower ratio of Bacteroidetes to Firmicutes in the gut of obese mice (Ley et al., 2005). In this study, a comparison between the HE and LE groups revealed a decreased ratio of Bacteroidetes to Firmicutes in the HE group, along with a significantly higher ADG in the HE group compared to the LE group. This suggests that there may be a negative correlation between the daily weight gain of beef cattle and the ratio of Bacteroidetes to Firmicutes. Research suggests that the ability to derive energy from the diet is greater in obese individuals and is linked to the gut microbiota structure (Turnbaugh et al., 2006). The altered rumen microbiota structure may be associated with the notably higher daily weight gain in the HE and ME groups compared to the LE group. In the rumen, Bacteroidetes primarily degrade carbohydrates and proteins. Research indicates that diets lower in calories but higher in fiber and protein favor the proliferation of Bacteroidetes (Guo et al., 2008). This is consistent with the results of this experiment, where the LE group had higher dietary fiber content than the ME and HE groups. *Prevotella* is essential in carbohydrate degradation, particularly in breaking down pectin, cellulose, and starch (Peng et al., 2015; Holman and Gzyl, 2019). The higher dietary cellulose content in the LE group compared to the ME and HE groups may explain the increased bacterial abundance of *Prevotella* in the rumen fluid of the LE group. Rikenellaceae is implicated in degrading plant-derived sugars, producing primarily propionic acid as a fermentation product (Ma et al., 2019). The rumen fluid of the HE group showed a significantly higher abundance of Rikenellaceae compared to the ME and LE groups, corresponding to a higher proportion of propionic acid. This indicates that the high-energy dietary level influenced rumen microbial composition, subsequently affecting rumen fermentation.

## 5 Conclusion

Increasing the comprehensive net energy level of the finishing diet for Angus steers to 7.990 MJ/kg (on a dry matter basis) leads to increased daily weight gain, a lower feed-to-gain ratio, and improved feed efficiency. Additionally, a short-term, high-intensity finishing regimen with a high net energy diet can increase intramuscular fat content, improve fatty acid composition, and enhance beef quality and nutritional value. However, prolonged feeding of high-energy diets may reduce microbial diversity in the rumen, negatively impacting intestinal health and potentially affecting the production performance of beef cattle.

## Data availability statement

The original contributions presented in the study are included in the article/supplementary material, further inquiries can be directed to the corresponding authors.

## Ethics statement

The animal study was approved by Administration of Laboratory Animals (CLI.2.293192, 2017 Revision, State Council, China) Institutional Animal Care and Use Committees of South China Agricultural University (Approval No. 2018-P002). The study was conducted in accordance with the local legislation and institutional requirements.

## Author contributions

KC: Data curation, Investigation, Methodology, Writing – original draft, Writing – review & editing. YS: Data curation, Methodology, Writing – review & editing. MD: Resources, Supervision, Writing – review & editing. YG: Resources, Supervision, Writing – review & editing. BS: Resources, Supervision, Writing – review & editing. GL: Resources, Supervision, Writing – review & editing. DL: Funding acquisition, Supervision, Writing – review & editing. YL: Funding acquisition, Supervision, Writing – review & editing.
